# Tracing the volatilomic fingerprint of grape pomace as a powerful approach for its valorization

**DOI:** 10.1016/j.crfs.2023.100608

**Published:** 2023-09-29

**Authors:** Teresa Abreu, Gonçalo Jasmins, Catarina Bettencourt, Juan Teixeira, José S. Câmara, Rosa Perestrelo

**Affiliations:** a CQM – Centro de Química da Madeira, Universidade da Madeira, Campus da Penteada, 9020-105, Funchal, Portugal; b Justino's Madeira Wines, S.A., Parque Industrial Da Cancela, Caniço, 9125-042, Santa Cruz, Portugal; c Departamento de Química, Faculdade de Ciências Exatas e Engenharia, Universidade da Madeira, Campus da Penteada, 9020-105, Funchal, Portugal

**Keywords:** Grape pomace, Volatile organic metabolites, HS-SPME/GC-MS, Circular economy, Valorization

## Abstract

The huge amount of grape pomace (GP) generated every year worldwide, particularly in Europe, creates negative impacts at the economic and environmental levels. As far as we know, scarce research has been done on the volatilomic fingerprint of GP. To meet consumer demand for healthy foods, there is a growing interest in the characterization of particular volatile organic metabolites (VOMS) in GP that can be used for industrial applications, including the food industry. In this study, the volatilomic fingerprint of GP obtained from different *Vitis vinifera* L. grapes was established by solid phase microextraction (HS-SPME) combined to gas chromatography-mass spectrometry (GC-MS), to explore the properties of most dominant VOMs in a context of its application on marketable products. A total of 52 VOMs belonging to different chemical families were identified. Alcohols, carbonyl compounds, and esters, are the most dominant, representing 38.8, 29.3, and 24.2% of the total volatile profile of the investigated GP, respectively. Esters (e.g., isoamyl acetate, hexyl acetate, ethyl hexanoate) and alcohols (e.g., 3-methyl butan-2-ol, hexan-1-ol) can be used as flavoring agents with potential use in the food industry, and in the cosmetic industry, for fragrances production. In addition, the identified terpenoids (e.g., menthol, ylangene, limonene) exhibit antioxidant, antimicrobial, and anticancer, biological properties, among others, boosting their potential application in the pharmaceutical industry. The obtained results revealed the potential of some VOMs from GP to replace synthetic antioxidants, colorants, and antimicrobials used in the food industry, and in the cosmetic and pharmaceutical industry, meeting the increasing consumer demand for natural alternative compounds.

## Highlights

• Establishment of the volatilomic fingerprint of grape pomace by HS-SPME/GC-MS.
• 52 volatile organic metabolites (VOMs) were identified in grape pomace.
• Alcohols, carbonyl compounds, and esters are the most dominant chemical families.
• The VOMs identified in GP revealed the potential to be used in industry applications.

## Graphical abstract

**Figure:**
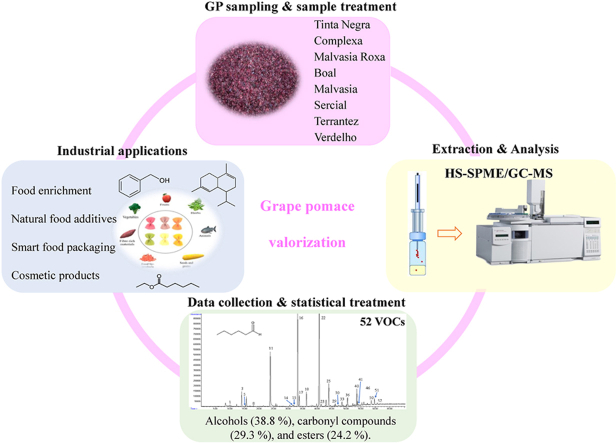

## 1 Introduction

**Fig. 1:**
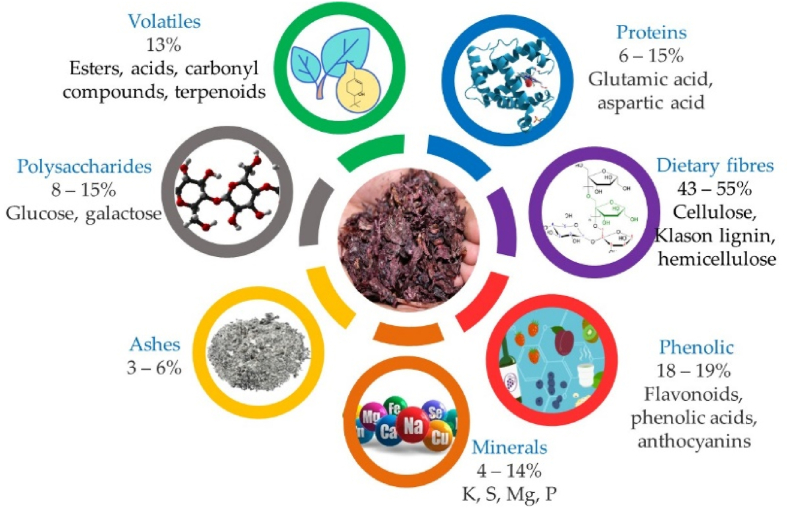
The main composition of grape pomace (composition expressed in %/100 g of dry material) [7].

Being one of the most important agricultural activities throughout the world, the winemaking processes generate huge quantities of under-utilized by-products and waste, namely GP generated by pressing whole grape bunches during the production of must, constituted of stalks, seeds, skins, and pulp, which account for 25–30% of produced grapes (Ferreira and Santos, 2022), from which grape skins are the major components contributing to half of the GP. This is especially important for Europe since about 65% of the world's wine production is managed by European winegrowers (Commission E., 2013) mainly from France, Italy, Spain, Germany, and Portugal. The improper disposal practices for winery wastes cause serious impacts on the environment, severely damaging the eco- and aqua-systems and, consequently, human and animal health (Câmara et al., 2020; Peixoto et al., 2018). However, GP is characterized by the presence of several high-value macromolecular components such as polysaccharides, proteins, and lipophilic compounds. In addition, the presence of high levels of bioactive compounds, such as phenolic compounds, anthocyanins, tannins, carotenoids, and dietary fibers (Fig. 1), further enhances its potential for industrial (food, pharmaceutic and cosmetic) applications. These compounds exhibit remarkably antimicrobial, anti-inflammatory, anticancer, antifungal, and antioxidant properties, establishing them as a valuable and sustainable bioresource (Ferreira and Santos, 2022; Ilyas et al., 2021; Peixoto et al., 2018) This approach will improve both the economic and environmental sustainability of the winemaking sector regarding its valorization based on the circular bioeconomy concept, constituting a powerful platform for shaping the future roadmap in the field of wine industry sustainability (Antonić et al., 2020; Bordiga et al., 2019; Jimenez-Lopez et al., 2020).

Nonetheless, most of the research on GP valorization has been conducted on phenolic compounds with the purpose of minimizing the waste's environmental burden and recovering extremely added-valued natural compounds. In this sense, Apostolou et al. (2013) verified that low dosages of proanthocyanidins from grape seeds suppress the proliferation of liver cervical (HeLa) and (HepG2) cells using *in vitro* settings. While Hamza et al. (2018) studied the anti-cancer property of grape seed extract and the data obtained demonstrated that this wine by-product has an anticancer impact by modifying oxidative damage, inducing apoptosis, inhibiting inflammatory response, and inhibiting cell growth. The anti-platelet activity of GP extracts, which plays a crucial role in atherothrombosis was evaluated by Choleva et al. (2019). Despite the significant and varied health benefits, the geometry and large molecular size of phenolic compounds are crucial for their bioavailability and bioaccessibility, making them more troublesome to use as therapeutic agents. In this sense, some VOMs such as terpenoids offer a better alternative for being low-molecular and lipophilic compounds that can penetrate the biological barriers with greater ease (Mahizan et al., 2019).

Nowadays, several headspace extraction processes, including dynamic headspace (DHS), solid phase microextraction (SPME), stir-bar sorptive extraction (SBSE), and gas chromatography (GC) combined with different detectors, have been employed to determine the volatile fingerprint of agri-food by-product (Câmara et al., 2020; Deng et al., 2021). From these, SPME is the most widely used headspace analysis for the extraction of VOMs because it offers a fully automated approach at a lower cost than other headspace techniques such as DHS and SBSE. In addition, SPME compared to traditional extraction procedures (liquid-liquid extraction, solid phase extraction) showed several advantages, like solvent-free sample preparation, a short extraction time, a small amount of sample required, and lower labor (Deng et al., 2021; Song et al., 2019). Generally, the SPME extraction procedure is coupled to gas chromatography with a sensitive mass spectrometry detector (GC–MS) and is highly used for the identification and quantification of several chemical families of VOMs in food-related products (Câmara et al., 2020; Song et al., 2019). In addition, GC-MS provides the identification of VOMs with a low limit of detection and the potential to separate complex mixtures.

As far as we know, scarce research has been done on the volatile fingerprint of GP. To meet consumer demand for healthy foods, there is a growing interest in the characterization of particular VOMs in GP that may be used in the food industry as antioxidant, antimicrobial, and anti-inflammatory active agents for the formulation of novel functional foods. In this context, the current study focused on the establishment of the volatile fingerprint of GP obtained from different *Vitis vinifera* L. grape varieties using HS-SPME followed by gas chromatography-mass spectrometry (GC-MS). To identify valuable VOMs that could be used in the food industry for the development of novel food ingredients and/or products for human consumption, pharmaceutical and cosmetic formulations, namely as antioxidant, antimicrobial, and anti-inflammatory agents. The discrepancies in the volatile signatures of the GP from different grape varieties under study were established using statistical methods.

## 2 Materials and methods

### 2.1 Chemicals

The Folin-Ciocalteu reagent (FR, 2 N), gallic acid monohydrate (≥98%), 2,2′-azino-bis-(3-ethylbenzothiazoline-6-sulfonic acid) diammonium salt (ABTS ≥98%), potassium persulfate (K_2_S_2_O_8_, 99%), trolox (C_14_H_18_O_4_, 98%), and 1,1-diphenyl-2-picrylhydrazyl (DPPH·≈ 90%) in free radical form were purchased from Fluka (Buchs, Switzerland). Anhydrous sodium carbonate (Na_2_CO_3_, 99.8%) was purchased from Panreac (Barcelona, Spain), and aluminum chloride anhydrous (AlCl_3_ 98%) from Riedel-de-Häen (Seelze, Germany). Hexanal (purity ≥98%), (*E*)-2-hexenal (≥98%), methyl acetate (99%), benzaldehyde (99.5%), ethyl propanoate (99%), isoamyl acetate (95%), methyl hexanoate (99%), ethyl decanoate (≥98%), ethyl benzoate (99%), toluene (99%), hexanoic acid (99%), acetic acid (99.9%), carvone (98%), caryophyllene (98.5%), limonene (≥95%), 2-phenyl ethanol (≥99%), benzyl alcohol (≥99%), hexan-1-ol (≥99.5%), (*E*)-3-hexen-1-ol (≥98%), (*Z*)-3-hexen-1-ol (≥98%), (*E*)-2-hexen-1-ol (≥95%), (*Z*)-2-hexen-1-ol (96%), 1-octen-3-ol (≥98%), heptan-1-ol (≥98%), 2-ethyl hexan-1-ol (≥99.6%) and octan-1-ol (≥99.9%) from Sigma-Aldrich (Madrid, Spain), whereas (*E*,*E*)-2,4-hexadienal (≥95%), benzeneacetaldehyde (≥95%) and menthol (≥95%) from Acros Organics (Geel, Belgium), and hexyl acetate (99%) and ethyl octanoate (99%) from Fluka (Buchs, Switzerland). These chemical standards were used to identify the target VOMs. Each VOM standard's stock solutions were dissolved in ethanol to a concentration of 500 mg/L and kept at 4 °C. Sigma-Aldrich (Madrid, Spain) provided the sodium chloride (NaCl, 99.5%) and 3-octanol (internal standard, IS, 99%), while Air Liquide (Portugal) supplied the helium (GC carrier gas) of purity 5.0. Supelco (Bellefonte, PA, USA) supplied the glass vials, divinylbenzene/carboxen/polydimethylsiloxane (DVB/CAR/PDMS) fiber, and SPME holder for manual sampling. Ultrapure water (H_2_O) (18 MΩ cm) was obtained from a Milli-Q water purification system (Millipore, Burlington, MA, USA). The alkane series, C8 to C20, with a concentration of 40 mg/L in n-hexane obtained from Fluka (Buchs, Switzerland) was used to determine the kovat index (KI).

### 2.2 Samples

Grapes were harvested upon ripening in the 2022 vintage and the GP was kindly provided by Justine Company (coordinates 32° 39′ 04″ North latitude and 16° 51′ 45″ West longitude) as a by-product of winemaking processes. The current research includes 3 red and 5 white *V. vinifera* L. grape varieties. The white grape varieties include Boal, Malvasia, Terrantez, Verdelho, and Sercial, while the red grape varieties include Malvasia Roxa (MR), Tinta Negra (TN), and Complexa. The GP from the different grape varieties was collected immediately after grapes pressing and transported in cool boxes under refrigeration (ca. 2–5 °C) to the laboratory. Afterward, the GP were lyophilized (Telstar, Cryodos, Spain) during 2 h, milled using a laboratory grinder (Grindomix GM200, Rech, Germany) to obtain a fine and homogeneous powder, and finally stored at − 80 °C until analysis.

### 2.3 Determination of total phenolics, total flavonoids, and antioxidant activity of GP extracts

To determine the total phenolic content (TPC), total flavonoids content (TFC) and antioxidant activity of grape pomace under study ethanolic extract were prepared. Briefly, 0.5 g of lyophilized grape pomace were placed in a vial with 3 mL of ethanolic solution 50% v/v. The mixture was then centrifuged for 5 min at 5000 rpm (centrifuge: SIGMA 1–7, max capacity 6 × 15 mL, max. RCF 6153×*g*) after being subjected to ultrasonic agitation for 5 min in an ultrasonic bath. After that, the supernatant was collected and submitted to spectrometric assays.

The TPC was determined spectrophotometrically using the Folin–Ciocalteu assay, while the TFC was estimated by the AlCl_3_ colorimetric assay according to Perestrelo et al. (2018). The results were expressed as milligrams of gallic acid equivalent per liter of sample [mg(GAE)/L] since gallic acid was utilized as a reference standard to plot the calibration curve. The spectrophotometric measurements were done with a UV–Vis spectrophotometer (Lambda 25, PerkinElmer, Waltham, MA, USA).

In vitro assays were carried out to evaluate the antioxidant activity of the free DPPH^●^ radical-scavenging capacity (A_AR_) and by the ABTS discoloring assay according to Perestrelo et al. (2018), with slight modifications. The DPPH method is based on the scavenging of DPPH^●^ by the addition of antioxidants able to discolor the DPPH solution which is proportional to the concentration of antioxidant type of molecules. For the DPPH^●^ radical assay, a stock solution of DPPH^●^ radical was prepared in ethanol (400 μM) and kept in the dark at 25 °C. For the reaction, the DPPH^●^ solution was diluted to obtain a working solution with an absorbance of 0.900(±0.030) at 515 nm. Trolox, used as a reference antioxidant, was assembled at concentrations ranging from 5 to 600 mg/L. The percentage of DPPH radical inhibition is calculated considering the absorbance before and after the reaction according to the following equation: %DPPH inhibition = [(A_0_-A_1_/A_1_) × 100], where A_0_ is the absorbance before the reaction, and A_1_ the absorbance after the reaction.

For the ABTS assay, a stock solution of ABTS (20 mM) was prepared in 50 mL of phosphate-buffered saline (PBS, pH 7.4), and 200 μL of 70 mM K_2_S_2_O_8_ solution (oxidant agent) was added. The solution was incubated in the dark at 25 ± 1 °C for 16 h. Then, diluted with PBS to obtain a working solution with an absorbance of 0.800 (±0.030) at 734 nm. After, an aliquot (12 μL) of each sample extract was mixed with 3 mL of ABTS^●+^ solution in 10 mm quartz cells and the decrease of absorption was measured at 734 nm for 30 min. The activity was expressed in terms of Trolox equivalents antioxidant capacity (TRE), for the analyzed extracts, plotting: ln(%ΔA_734_) vs Trolox concentration (from 5 to 600 mg/L). All determinations were carried out in triplicate.

### 2.4 HS-SPME procedure

The SPME method employed in this study was adapted from earlier research projects conducted in our laboratory (Câmara et al., 2020). Briefly, 2 g of each lyophilized GP, 0.5 g NaCl (salting-out effect), 5 μL of 3-octanol (IS, 250 μg/L), and 5 mL of H_2_O were added in a 20 mL glass vial containing a stirring bar (2 × 0.5 mm, 600 rpm). After that, the glass vial was capped with PTFE-coated silicone septum and placed in a thermostatic block at 40 °C and the DVB/CAR/PDMS fiber was inserted into the headspace sample vial for 45 min. After the VOMs extraction, the fiber was withdrawn into the needle holder, removed from the vial, and placed in the GC injector at 250 °C for 6 min for thermal desorption of VOMs. All analyses were performed in triplicate (n = 3). Before practice, the SPME fiber was thermally conditioned following the manufacturer's recommendations. Each day, conditioning was performed for 10 min prior to the first extraction to verify the lack of carryover analytes. Moreover, a blank was carried out between samples to confirm the lack of carryover analytes.

### 2.5 GC-MS analysis conditions

Chromatographic separation of the VOMs was done using an Agilent 6890 N (Palo Alto, CA, USA) gas chromatography system equipped with BP-20 fused silica (30 m × 0.25 mm inner diameter × 0.25 μm thick) capillary column purchased from SGE (Darmstadt, Germany). Helium was used as carrier gas with a flow rate of 1 mL/min. The temperature of the injector, GC-qMS interface, and quadrupole detector were 250, 180, and 220 °C, respectively. The GC oven temperature started at 45 °C (1 min), increased to 100 °C at 2 °C/min (3 min), followed by an increase to 130 °C at 5 °C/min (5 min) reaching the final temperature of 220 °C at 7 °C/min (15 min), total run time of 70.35 min. The MS used was an Agilent 5975 quadrupole inert mass selective detector, which operated on electron impact (EI) at 70 eV, maintaining the source temperature at 180 °C. The mass range acquired was from 30 to 300 *m*/*z*, at the rate of 1.9 spectra/s, in full scan mode. The VOMs were identified by comparison of the GC retention times (RT), kovat index (KI) and mass spectra with those of the standard, when available; and by comparison of their mass spectra with those in the National Institute of Standards and Technology (NIST) MS 05 spectral database (Gaithersburg, MD, USA) with a matching probability >85%. The van den Dool and Kratz equation was used to determine the KI values, which were then compared to values reported in the literature for equivalent columns (El-Sayed, 2011).

The relative-quantification of VOMs was done using 3-octanol (IS) addition based on the following equation: VOMs concentration = (VOM GC peak area/IS GC peak area) × IS concentration.

### 2.6 Data treatment and statistical analysis

A chemical network containing all VOMs (except the ones common to all samples) was constructed by Gephi (version 0.9.7), a network-building tool. Excel was used to transform and import the data to Gephi. This data is comprised of two parts, the nodes, and edges. The nodes correspond to the samples and VOMs, while the edges establish a connection between the nodes (Adam et al., 2021). The antioxidant test values were normalized in an interval between 30 and 150 to better visualize the sample differences in Gephi according to Polatgil et al. (Polatgil, 2022). This interval was attained by trial and error. TPC and TFC were normalized together (sharing the same max(x) and min(x)), while ABTS and DPPH values were normalized independently since they have a different order of magnitude.

The statistical analysis was carried out using the MetaboAnalyst 5.0 web-based tool (Pang et al., 2021). The raw GC-MS data were pre-processed to eliminate VOMs with miss values and then normalized by Log transformation (base 10) and auto-scaling (mean-centered and divided by the standard deviation of each variable). The dataset was subjected to a one-way analysis of variance (ANOVA) followed by Fisher's test for post-hoc multiple comparisons of means of GP from white and red grape varieties data at p_value_ < 0.05 to recognize statistically significant. Principal component analysis (PCA) was used to reduce the dimensionality of the dataset and to obtain a representation from the data projection. Partial least squares-discriminant analysis (PLS-DA), a supervised method based on PLS regression, was applied to the GP volatile fingerprint dataset to provide insights into the separations between GP from white and red grape varieties and to identify VOMs that contribute to differentiate among sample sets, therefore useful to discriminate variable selection.

## 3 Results and discussion

### 3.1 Assessment of the antioxidant potential of grape pomace

**Fig. 2:**
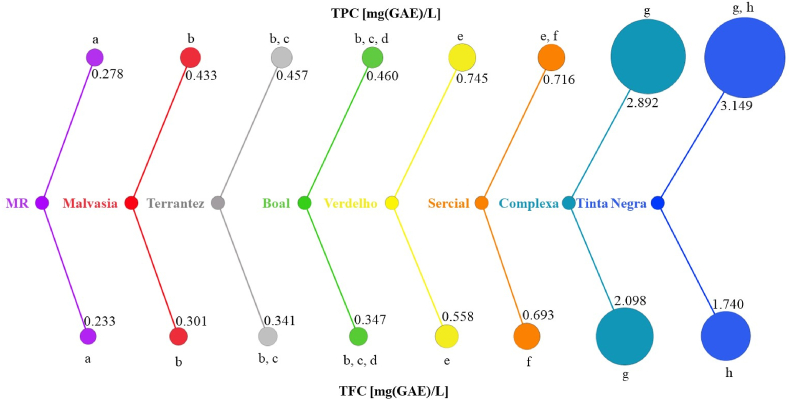
Total phenolic content (TPC) and total flavonoid content (TFC) of GP from different *V. vinifera* L. grapes. Different superscript letters suggest significant differences (p < 0.05) in TPC and TFC levels in GP from different grape varieties studied (MR: Malvasia Roxa).

The antioxidant potential of the GP from studied grape varieties was evaluated through spectrophotometric assays (TPC, TFC, DPPH, ABTS). Due to their redox characteristics, phenolic compounds are known to have strong antioxidant activity and play significant roles in absorbing and neutralizing free radicals (Perestrelo et al., 2018). Fig. 2 displays the TPC and TFC patterns of GP from white (Boal, Malvasia, Terrantez, Verdelho, and Sercial) and red (Tinta Negra, Malvasia Roxa, Complexa) *V. vinifera* L. grape varieties.

As can be observed, significant differences (p < 0.05) were verified for TPC and TFC amounts among the GP of the investigated grape varieties, except for GP from Boal and Terrantez varieties. GP from Tinta Negra showed the highest TPC amount (3.15 ± 0.02 mg/L), followed by Complexa (2.89 ± 0.06 mg/L), Verdelho (0.75 ± 0.02 mg/L), Sercial (0.72 ± 0.01 m/L), Boal (0.46 ± 0.01 mg/L) ∼ Terrantez (0.46 ± 0.01 mg/L), Malvasia (0.43 ± 0.01 mg/L), and Malvasia Roxa (0.28 ± 0.01 mg/L). On the other hand, the highest TFC amount (2.10 ± 0.04 mg/L) was determined in GP of the Complexa grape variety, while the lowest concentration was found in Malvasia Roxa (0.23 ± 0.01 mg/L).

**Fig. 3:**
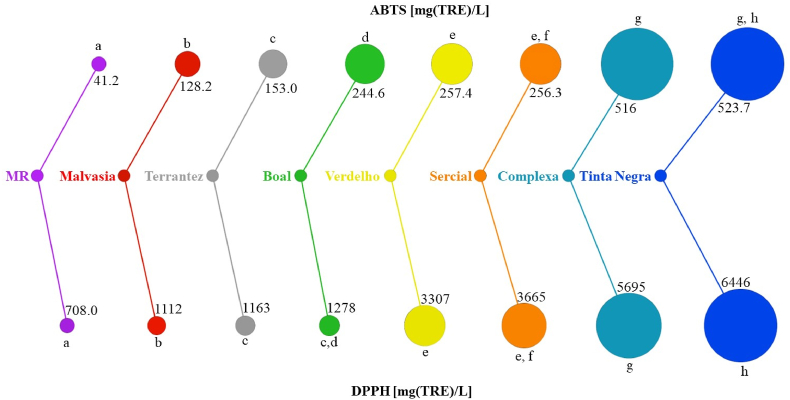
DPPH scavenging and ABTS assays of grape pomace from different *V. vinifera* L. grapes. Different superscript letters suggest significant differences (p < 0.05) among GP under study.

The antioxidant potential of the GP under investigation was assessed spectrophotometrically through DPPH and ABTS assays. The results are shown in Fig. 3. The GP from Tinta Negra (524 ± 16.4 and 6446 ± 31.6 mg/L for ABTS and DPPH assays, respectively) and Complexa (516 ± 24.3 and 5695 ± 18.8 mg/L) showed antioxidant activity significantly higher than the obtained for the other grape varieties, while Malvasia Roxa (42.1 ± 4.84 and 708 ± 10.6 mg/L) is the grape variety with the lowest antioxidant capacity, considering the GP from grape varieties under study. In addition, no significant difference (p < 0.05) was observed in the antioxidant capacity of GP from Sercial (256.3 ± 10.5 and 3665 ± 34.8 mg/L for ABTS and DPPH assays, respectively) and Verdelho (257.4 ± 13.2 and 3307 ± 31.7 mg/L).

In sum, the antioxidant potential is highest in GP from red grapes compared to white grape varieties, except for Malvasia Roxa. Although Malvasia Roxa is a red *V. Vinifera* L. grape variety, its antioxidant capacity is close to that determined in white *V. Vinifera* L. grape varieties.

### 3.2 Volatile fingerprint of grape pomace

To valorize the GP from red and white grape varieties, a valuable source of bioactive compounds with several biological properties (e.g., antioxidant, antimicrobial, antiproliferative), a volatilomic-based analytical approach was carried out. The goal was to establish the volatile fingerprint of GP from different *V. vinifera* L. grapes, aiming to explore and identify some VOMs with potential industrial applications in the fields of pharmaceuticals, cosmetics, and food. A total of 52 VOMs were identified, from which 18 were common to all analyzed samples. The identified VOMs belong to different chemical families including alcohols (14), esters (14), carbonyl compounds (10), terpenoids (11), acids (2), and hydrocarbon (1) (Table 1).

**Table 1:** Relative concentration (μg/L) and relative standard deviation (in brackets) of volatile organic metabolites identified in grape pomace.

Peak nº	RT (min)	KI_cal_^a^	KI_lit_^b^	Chemical families	Identification^c^	Tinta Negra	Complexa	Malvasia Roxa	Boal	Malvasia	Sercial	Terrantez	Verdelho
				**Carbonyl compounds**									
1	10.0	679	690	Acetaldehyde	NIST (89%)	0.84 (16)	0.04 (9)	0.03 (16)	0.17 (12)	0.64 (10)	0.03 (7)	0.20 (7)	0.07 (12)
4	15.0	902	906	2-Methylbutanal	S	–	0.02 (14)	–	–	0.16 (15)	–	0.09 (7)	–
5	15.1	910	917	3-Methylbutanal	NIST (94%)	–	0.03 (5)	–	0.04 (10)	0.25 (4)	–	0.07 (20)	0.07 (9)
8	18.0	972	979	Pentanal	NIST (87%)	0.19 (14)	0.03 (16)	0.05 (5)	0.09 (13)	0.19 (8)	0.03 (19)	0.03 (6)	0.06 (13)
11	23.8	1044	1048	Hexanal	S	2.61 (15)	2.00 (2)	0.64 (18)	6.26 (2)	6.16 (14)	0.70 (17)	1.09 (2)	3.64 (14)
16	33.3	1210	1205	(*E*)-2-Hexenal	S	0.19 (8)	3.00 (10)	3.34 (19)	8.60 (7)	8.53 (10)	2.89 (7)	–	8.42 (11)
21	40.0	1322	1322	6-Methyl-5-hepten-2-one	NIST (86%)	0.09 (8)	0.04 (11)	0.03 (14)	0.07 (15)	0.14 (12)	0.03 (3)	0.08 (20)	0.07 (4)
27	44.8	1410	1405	(*E*,*E*)-2,4-Hexadienal	S	0.03 (19)	0.10 (14)	–	0.08 (4)	0.21 (6)	0.05 (4)	–	–
36	50.4	1479	1486	Benzaldehyde	S	0.26 (9)	0.14 (8)	0.07 (11)	0.70 (16)	0.19 (8)	0.12 (17)	0.25 (5)	0.14 (3)
41	54.2	1669	1656	Benzeneacetaldehyde	S	–	0.11 (4)	0.06 (9)	0.15 (14)	0.32 (12)	0.11 (4)	0.05 (4)	–

				**Esters**									
2	12.3	810	813	Methyl acetate	S	0.11 (18)	0.10 (11)	0.05 (11)	0.20 (8)	0.17 (11)	0.13 (19)	0.19 (21)	0.27 (13)
3	14.0	871	874	Ethyl acetate	NIST (94%)	21.1 (1)	0.35 (15)	0.23 (8)	1.54 (1)	3.29 (12)	1.29 (11)	47.3 (12)	2.38 (7)
6	16.9	952	955	Ethyl propanoate	S	0.14 (10)	0.02 (10)	0.02 (12)	0.04 (14)	–	0.02 (12)	0.08 (15)	0.03 (9)
7	17.6	965	970	Propyl acetate	NIST (95%)	0.06 (16)	–	–	–	–	–	0.18 (11)	–
9	19.5	994	993	Isobutyl acetate	NIST (97%)	0.22 (17)	0.02 (9)	–	0.03 (13)	–	0.03 (9)	0.14 (5)	–
12	26.1	1098	1102	Isoamyl acetate	S	1.75 (8)	0.02 (5)	0.02 (1)	0.14 (14)	0.14 (14)	0.02 (6)	6.32 (15)	0.07 (7)
13	30.6	1163	1154	Methyl hexanoate	S	0.04 (7)	0.02 (12)	–	0.16 (3)	–	0.02 (9)	–	–
17	33.8	1217	1212	Ethyl hexanoate	S	0.85 (15)	0.39 (15)	0.14 (5)	0.96 (2)	–	0.10 (9)	0.62 (18)	0.10 (2)
18	36.3	1268	1251	Hexyl acetate	S	1.74 (19)	0.22 (6)	0.06 (10)	1.24 (10)	1.84 (17)	0.94 (8)	4.59 (14)	2.11 (16)
20	39.0	1315	1321	3-Hexen-1-ol acetate	NIST (98%)	0.38 (7)	–	–	0.02 (6)	0.36 (1)	0.05 (11)	0.11 (4)	0.08 (16)
28	45.7	1439	1433	Ethyl octanoate	S	1.47 (21)	0.04 (9)	0.04 (5)	–	0.23 (12)	0.02 (5)	0.10 (13)	0.13 (12)
39	53.2	1615	1609	Ethyl decanoate	S	0.29 (9)	–	0.02 (20)	0.06 (19)	0.14 (6)	–	–	0.04 (2)
42	54.8	1672	1662	Ethyl benzoate	S	0.08 (4)	–	–	0.06 (11)	–	–	–	–
49	58.5	1832	1823	2-Phenylethyl acetate	S	0.91 (13)	–	0.06 (17)	0.09 (16)	–	–	0.47 (5)	–

				**Alcohols**									
14	31.5	1197	1196	3-Methyl butan-2-ol	NIST (94%)	8.18 (2)	0.05 (17)	0.02 (2)	0.16 (2)	0.86 (16)	0.04 (18)	6.97 (16)	0.14 (19)
19	38.5	1279	1280	Heptan-2-ol	NIST (89%)	0.02 (13	0.02 (17)	0.03 (13)	–	–	0.02 (10)	–	0.08 (14)
22	40.6	1334	1324	Hexan-1-ol	S	15.8 (11)	4.29 (7)	1.33 (17)	17.1 (12)	25.5 (6)	3.49 (11)	9.32 (2)	6.60 (7)
23	41.4	1346	1349	(*E*)-3-Hexen-1-ol	S	0.58 (11)	0.03 (14)	0.02 (15)	0.18 (7)	0.21 (8)	0.04 (5)	0.03 (4)	0.11 (7)
24	42.8	1375	1377	(*Z*)-3-Hexen-1-ol	S	0.02 (11)	0.12 (17)	0.11 (4)	0.25 (10)	2.16 (16)	0.17 (13)	0.14 (11)	0.11 (3)
25	44.0	1390	1388	(*E*)-2-Hexen-1-ol	S	0.98 (11)	0.21 (6)	0.45 (15)	2.16 (2)	2.98 (13)	0.27 (7)	0.12 (19)	0.37 (7)
26	44.7	1408	1410	(*Z*)-2-Hexen-1-ol	S	–	–	0.07 (12)	0.07 (15)	0.23 (6)	0.04 (10)	–	0.13 (15)
29	46.2	1444	1437	1-Octen-3-ol	S	5.13 (17)	0.03 (10)	0.02 (20)	0.16 (17)	0.55 (11)	0.02 (13)	0.02 (2)	0.05 (17)
30	46.4	1450	1456	Heptan-1-ol	S	0.21 (11)	0.09 (8)	–	0.07 (6)	0.07 (11)	–	0.07 (4)	0.07 (9)
32	47.7	1483	1484	2-Ethyl hexan-1-ol	S	–	0.05 (9)	0.06 (3)	0.07 (10)	–	0.05 (4)	–	0.12 (19)
37	50.6	1502	1517	Octan-1-ol	S	0.13 (11)	0.10 (10)	0.03 (6)	0.11 (12)	0.20 (5)	0.02 (6)	0.08 (16)	–
44	55.3	1692	1705	Undecanol	NIST (87%)	0.18 (13)	–	–	0.12 (1)	–	–	–	0.13 (9)
51	59.7	1915	1902	Benzyl alcohol	S	0.28 (11)	0.35 (14)	0.09 (19)	0.61 (1)	0.30 (13)	0.08 (2)	0.25 (17)	0.15 (9)
52	60.6	1966	1965	2-Phenyl ethanol	S	2.80 (11)	0.12 (10)	0.11 (9)	0.25 (7)	0.39 (11)	0.14 (2)	1.78 (12)	0.36 (7)

				**Terpenoids**									
15	31.7	1199	1197	Limonene	S	0.21 (15)	–	–	0.09 (7)	–	0.01 (13)	–	–
33	48.5	1499	1491	Ylangene	NIST (85%)	0.14 (11)	0.65 (16)	0.04 (4)	0.35 (1)	0.41 (6)	0.02 (7)	–	–
34	49.5	1540	1544	β-Bourbonene	NIST (88%)	0.04 (12)	0.04 (8)	0.05 (14)	0.11 (5)	–	–	0.03 (11)	–
35	50.2	1476	1490	(*E*)-Limonene oxide	NIST (87%)	0.17 (6)	0.11 (10)	0.07 (8)	–	0.17 (17)	–	0.11 (14)	0.14 (9)
38	52.6	1602	1611	Caryophyllene	S	0.11 (8)	–	0.04 (11)	0.06 (16)	0.12 (14)	–	–	0.04 (9)
40	53.6	1629	1631	Menthol	S	3.64 (6)	0.36 (10)	0.30 (4)	0.72 (6)	2.18 (12)	0.14 (17)	1.84 (12)	–
43	54.9	1673	1663	α-Caryophyllene	NIST (86%)	0.09 (9)	–	–	0.06 (8)	0.12 (7)	–	–	–
45	55.7	1697	1688	Epizonarene	NIST (88%)	0.03 (12)	0.07 (11)	–	0.05 (8)	0.08 (7)	–	–	–
46	55.8	1699	–	Isoledene	NIST (85%)	0.04 (9)	0.06 (5)	–	0.12 (13)	0.65 (15)	–	–	–
47	56.6	1745	1740	Carvone	S	0.12 (16)	0.09 (3)	–	–	0.16 (16)	–	–	0.09 (15)
48	57.0	1802	1815	α-Cadinene	NIST (87%)	–	–	–	0.10 (6)	0.13 (15)	–	–	–

				**Acids**									
31	47.0	1466	1463	Acetic acid	S	3.87 (17)	0.02 (7)	0.04 (5)	0.18 (12)	2.24 (15)	–	2.57 (6)	0.16 (7)
50	58.7	1836	1824	Hexanoic acid	S	0.51 (19)	0.19 (14)	0.32 (6)	0.53 (4)	1.39 (3)	0.17 (4)	0.56 (5)	–

				**Benzene derivates**									
10	21.5	1032	1034	Toluene	S	0.10 (3)	0.04 (13)	0.05 (4)	0.07 (12)	–	0.03 (14)	0.33 (14)	–

a Kovats index relative n-alkanes (C8 to C20) on a BP-20 capillary column.

b Kovats index relative reported in literature for equivalent capillary column [15].

c Identification confirmed by comparing mass spectra and retention time with those of authentic standard (S), and by NIST library (matching probability %). -: not detected.

**Fig. 4:**
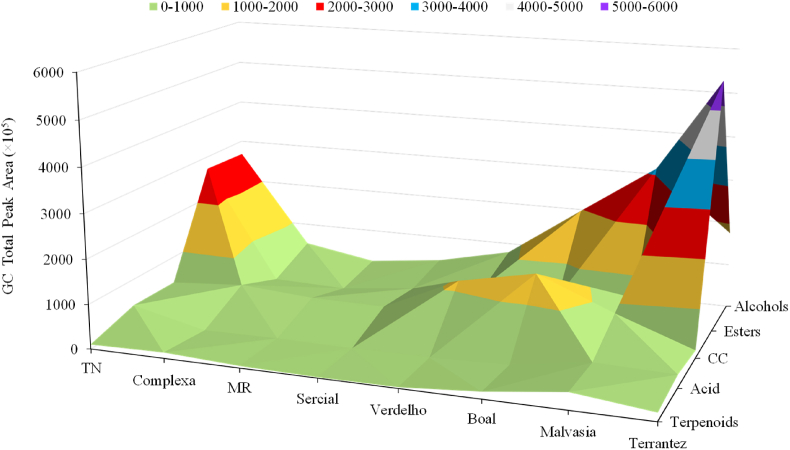
The total peak area of chemical families identified in GP of investigated grape varieties. MR: Malvasia Roxa, CC: Carbonyl compounds.

Fig. 4 demonstrates the total peak area of each chemical families identified in GP from the investigated *V. vinifera* L. grape varieties. In terms of total GC peak area, alcohols are the most abundant chemical (on average 36.9 ± 1.85 and 39.9 ± 2.79% of the total volatile fraction of GP from red and white grapes, respectively), followed by carbonyl compounds (32.7 ± 2.27 and 27.2 ± 1.09%), esters (19.9 ± 1.56 and 26.7 ± 1.34%), terpenoids (5.92 ± 0.18 and 3.16 ± 0.29%), acids (4.16 ± 0.38 and 2.76 ± 0.14%) and hydrocarbon (0.34 ± 0.02 and 0.17 ± 0.01%).

The alcohol chemical family is composed of aromatic (e.g., 2-phenyl ethanol, benzyl alcohol) and aliphatic (e.g., 3-methyl butan-2-ol, hexan-1-ol) volatiles. The GC total peak area of alcohols is, on average, 0.79 times higher in GP obtained from red grapes than the white grapes. Hexan-1-ol (on average, the relative concentration was 7.15 ± 0.74 and 10.3 ± 0.62 μg/L in GP from red and white grapes, respectively), 3-methyl butan-2-ol (2.75 ± 0.06 and 1.36 ± 0.22 μg/L), (*E*)-2-hexen-1-ol (0.54 ± 0.03 and 0.98 ± 0.08 μg/L), and 2-phenyl ethanol (1.01 ± 0.10 and 0.49 ± 0.05 μg/L) are the most abundant alcohols. These chemical families can impact a strong, unpleasant smell and taste at concentrations higher than 400 mg/L, but at concentrations lower than 300 mg/L, they can enhance the overall aroma with fruit notes (Zhang et al., 2020). 2-Phenyl ethanol is associated with floral and honey odors, 3-methyl butan-2-ol to banana, 1-hexanol, and (*E*)-2-hexen-1-ol to green/herbaceous odors.

Carbonyl compounds (ketones and aldehydes) are the second most abundant chemical family in the analyzed GP. Their contribution to the total volatile fingerprint of GP from the different grape varieties is significantly different. On average, the highest GC total peak area was determined in Malvasia Roxa (51.9%), followed by Verdelho (47.0%), Complexa (40.1%), Boal (36.5%), Sercial (35.2%), Malvasia (15.4%), Tinta Negra (6.25%) and Terrantez (2.16%). Hexanal and 2-hexenal are the most dominant aldehydes in the GP understudy, accounting for 82.9 and 87.9% of the total fraction of carbonyl compounds in GP from red and white grapes varieties under investigation.

The contribution of esters to the total volatilomic fingerprint ranges from 8.05 (Malvasia Roxa) to 69.7% (Terrantez). The highest esters contribution for GP of Terrantez (69.7%) and Tinta Negra (43.1%) grape varieties is explained by the high relative concentration of ethyl acetate, 21.1 ± 0.20 and 47.3 ± 5.65 μg/L, respectively. Nevertheless, the esters' contributions in GP of Malvasia Roxa (8.046%) and Complexa (8.6%) are quite similar. The esters contribute positively with aromas and flavors common to fruits, such as bananas, strawberries, pineapple, raspberries, cherries, and even citrusy and floral odors. In general, esters have low odor thresholds (trace μg/L) depending on the matrix (Perestrelo et al., 2020). The presence of esters in GP is scarce since the vast majority of esters that occur in wine are produced during fermentation. Isoamyl acetate, hexyl acetate, ethyl hexanoate, and ethyl octanoate are, on average, the most abundant esters identified in GP from investigated grape varieties. It should be noted that the GC peak area of isoamyl acetate and hexyl acetate are, on average, 1.87 and 2.66 times higher in GP from white grapes than from red varieties, while ethyl hexanoate and ethyl octanoate are, on average, 1.20 and 4.88 times higher in GP from red grapes than white grapes.

**Fig. 5:**
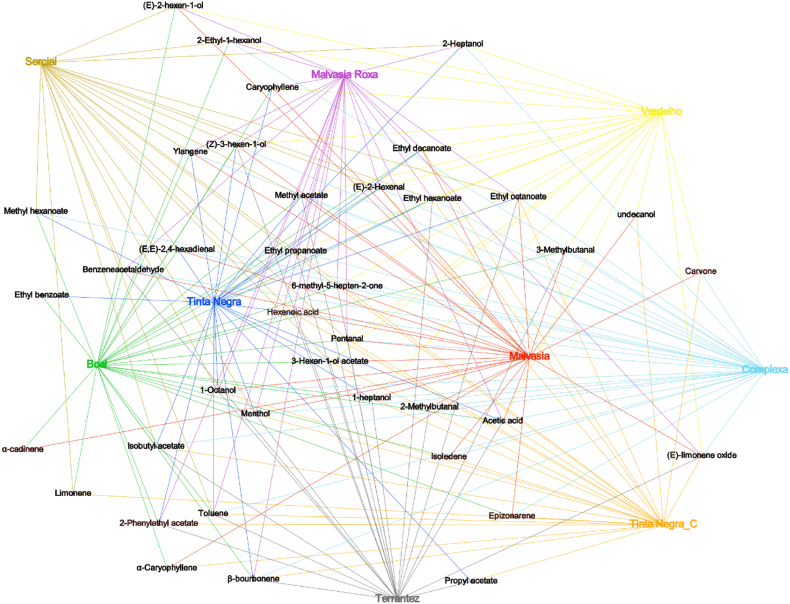
Volatilomics network of the investigated GP.

In terms of total relative concentration, Malvasia seems to be the richest grape variety in terpenoids (4.02 ± 0.48 μg/L), followed by Terrantez (1.98 ± 0.23 μg/L), Boal (1.67 ± 0.10 μg/L), Complexa (1.38 ± 0.16 μg/L), Tinta Negra (1.11 ± 0.15 μg/L), Malvasia Roxa (0.50 ± 0.03 μg/L), Verdelho (0.27 ± 0.03 μg/L), and Sercial (0.18 ± 0.03 μg/L). The total relative concentration of terpenoids determined in white grape varieties was, on average, 1.63 times higher than in GP from red varieties. This chemical family could be used as potential molecular markers since some of the terpenoids identified are only detected in the GP of some grape varieties under investigation (Fig. 5). For example, α-amorphene is only detected in GP from Boal and Malvasia grapes, limonene from Tinta Negra, Boal and Sercial grapes, and α-caryophyllene in GP from Tinta Negra, Boal, and Malvasia grapes. On the other hand, menthol was detected in the GP of all grape varieties investigated, except for GP obtained from Verdelho grapes.

### 3.3 Bioactive potential of volatile organic metabolites identified in grape pomace and potential applications

Several VOMs identified in GP from red and white grape *V. vinifera* L. varieties under study have been previously reported in food and food-related products with a remarkable number of biological properties, namely as antioxidant, antimicrobial, anti-inflammatory, antiproliferative, antidiabetic, antitumor activities, boosting their applications in different fields including in the food industry, as preservatives, additives, and flavoring agents, in the pharmaceutical industry as anti-inflammatory antiproliferative, and antidiabetic agents, and also in the cosmetic industry as flavor and coloring agents (Alam et al., 2019; Assmann et al., 2018; Dănilă et al., 2018; Elkady and Ayoub, 2018).

As previously discussed, alcohols and esters are the chemical families that most contribute to the total volatile profile of grape pomace, independently of grape varieties. Esters (e.g., isoamyl acetate, hexyl acetate, ethyl hexanoate) and alcohols (e.g., benzyl alcohol, 2-phenyl ethanol) are commonly used as flavoring agents in various food products such as candies, baked goods, and beverages; and as ingredients in perfumes, soaps, and some others personal care products (De Melo Pereira et al., 2019). Moreover, isoamyl acetate, 3-methyl butan-2-ol, benzyl alcohol, and 1-octen-3-ol have also been shown to have antimicrobial activity against several microorganisms (Ando et al., 2015; Xiong et al., 2017). In addition, Inamdar et al. (2014) explored the signaling pathways involved in 1-octen-3-ol-mediated dopamine neurotoxicity, and the obtained data showed that 1-octen-3-ol exposure was associated with activation of caspase 3, a lysosomal enzyme (endoprotease) involved in the apoptotic pathway. In another study, 2-ethyl hexan-1-ol and 2-pheny ethanol have been reported in fish feeds (Giogios et al., 2009), whereas a mixture of 3-hexenol and 2-hexenal induce light stress-alleviating effects on dog (Carlone et al., 2018). Regarding carbonyl compounds, special attention has been devoted to hexanal and benzaldehyde, which are powerful antimicrobial constituents because of their capacity to interact with the sulfhydryl and amino groups of protein moieties in the microbial cytoplasmic membrane, causing the membrane transport function to be disrupted and ultimately leading to cell death. In addition, these additives are classified as generally recognized as safe (GRAS) status, being used to add flavor to foods, and a potent inhibitor of phospholipase D activity (Jash et al., 2018; Ranjan et al., 2020). Hexanal application produced positive results by extending the shelf life of a diversity of fruits (Anusuya et al., 2016; Debysingh et al., 2018; Ranjan et al., 2020). On the other hand, terpenoids are present in the GP of some studied grapes but in lesser amounts. However, their total GC peak areas are higher in GP from white grapes than in red grapes. Menthol and ylangene are the most dominant terpenoids. Some studies have reported that menthol possesses anti-inflammatory, antibacterial, antitumor, antiviral, scolicidal, immunomodulatory, neuroprotective, antifatigue, and antioxidant activities (Zhao et al., 2022), while ylangene is related to cytotoxic and anti-inflammatory properties (Xio et al., 2013). In addition, terpene-mediated actions have been found to be protective against tuberculosis by Sieniawska et al. (2018). Nevertheless, the most noteworthy bioactivity of terpenoids is related to their anticancer potential, acting at different stages of tumor development and in different mechanisms of action (inhibition, regulation of intracellular signaling pathways) (Ansari and Akhtar, 2019; Cho et al., 2017). Numerous studies have proven limonene's chemopreventive and chemotherapeutic effects against several human malignancies (Chebet et al., 2021; Zhou et al., 2021).

### 3.4 Statistical analysis

**Fig. 6:**
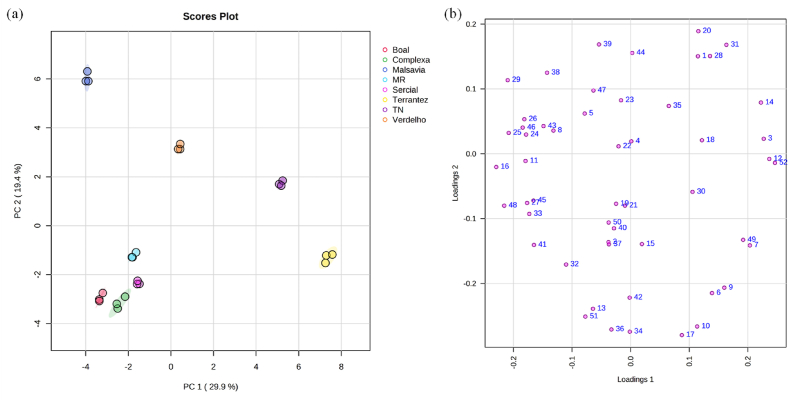
PCA of the volatile fingerprint of GP. (a) PC1 × PC2 score scatter plot and (b) loading weight plot (attribution of the peak number is pointed out in Table 1).

Volatile fingerprint was evaluated to discover potential similarities/differences for discrimination between the GP from white and red *V. vinifera* L. grape varieties. The one-way ANOVA with post-hoc Tukey test (p < 0.05) demonstrated that the p_values_ obtained evidence that the identified 52 VOMs presented statistically significant differences for the analyzed GP. Principal component analysis (PCA), a multivariate unsupervised pattern recognition technique, was used to pre-process and reduce the dimensionality of the dataset while preserving the structure inherent to the initial matrix. The dataset composed of 52 VOMs and 24 samples (eight samples × three collections) was submitted to PCA. Fig. 6 (a) and (b) displayed the PCA score plot and loading plot from the GP respectively.

**Fig. 7:**
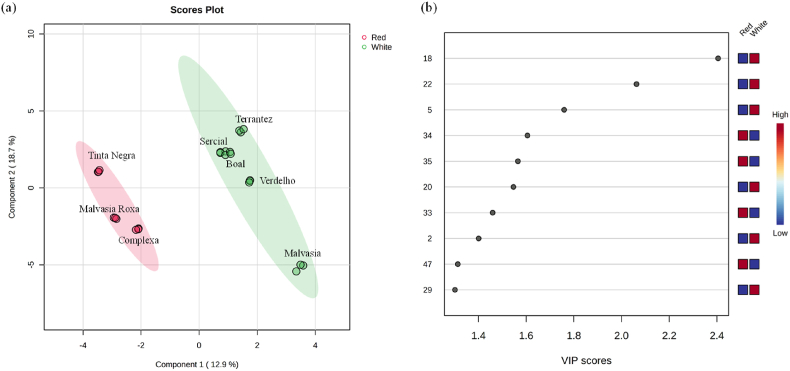
PLS-DA of the volatile fingerprint of grape pomace (a) score scatter plot, and (b) VIP scores. Number identification: (2) methyl acetate; (5) 3-methylbutanal; (18) hexyl acetate; (20) 3-hexen-1-ol acetate; (22) hexan-1-ol; (29) 1-octen-3-ol; (33) ylangene; (34) β-bourbonene; (35) (*E*)-limonene oxide; (47) carvone.

The first (PC1) and the second (PC2) principal components account for 49.3% of the total explained variance suggesting that 50.7% of the variables distributes across more than one dimension. Tinta Negra and Verdelho GP, projected in PC1 and PC2 positive quadrant, are mainly associated with hexyl acetate (18), heptan-1-ol (30) and (*E*)-limonene oxide (35), whereas the GP obtained from Boal, Sercial, Malvasia Roxa, and Boal are projected in PC1 and PC2 negative being correlated to heptan-2-ol (19), 2-ethyl hexan-1-ol (32), menthol (40), benzeneacetaldehyde (41), and hexanoic acid (50). Malvasia GP, placed in PC1 negative and PC2 positive, was mainly characterized by 1-octen-3-ol (29) and caryophyllene (38), while Terrantez GP projected in PC1 positive and PC2 negative to propyl acetate (7) and 2-phenylethyl acetate (49). Moreover, partial least square-discriminant analysis (PLS-DA) was used and the ten most significant VOMs (VIP score >1) that allowed the discrimination of the GP by *V. vinifera* L. variety were methyl acetate (2), 3-methylbutanal (5), hexyl acetate (18), 3-hexen-1-ol acetate (20), hexan-1-ol (22), 1-octen-3-ol (29), ylangene (33), β-bourbonene (34), (*E*)-limonene oxide (35), and carvone (47) as can be seen in Fig. 7b.

**Fig. 8:**
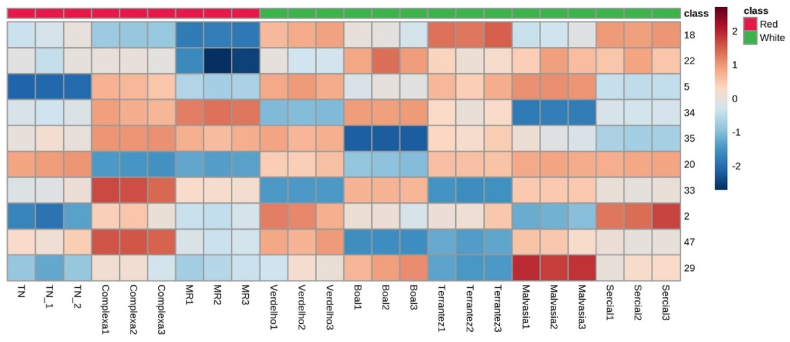
Hierarchical cluster analysis (HCA) of volatile fingerprint of grape pomace. Number identification: (2) methyl acetate; (5) 3-methylbutanal; (18) hexyl acetate; (20) 3-hexen-1-ol acetate; (22) hexan-1-ol; (29) 1-octen-3-ol; (33) ylangene; (34) β-bourbonene; (35) (*E*)-limonene oxide; (47) carvone.

The heatmap created using Pearson's correlation for the VOMs with VIP scores >1 is shown in Fig. 8. The heatmap shows that the ylangene (33), carvone (47), and in lower extension 3-methylbutanal (5), β-bourbonene (34), 3-hexen-1-ol acetate (20) and (*E*)-limonene oxide (35) are positively associated with GP from red grapes. These GP are negatively associated with hexyl acetate (18), hexan-1-ol (22), methyl acetate (2), and 1-octen-3-ol (29). The GP from white grapes is strongly associated with 1-octen-3-ol (29), methyl acetate (2) and hexyl acetate (18), and in the lower extent to hexan-1-ol (22), 1-octen-3-ol (29), and 3-hexen-1-ol acetate (20), depending on the grape varieties. This variation may be the consequence of environmental conditions (e.g., mean annual temperature), geographic location (e.g., longitude, latitude), which may limit the activity of specific VOMs, as well as the practices used throughout the winemaking process (Perestrelo et al., 2014).

## 4 Conclusions

The HS-SPME/GC-MS analytical methodology was used in the current research with the purpose to identify VOMs in GP from different *V. vinifera* L. grape varieties as a useful strategy to investigate the potential properties of the dominant VOMs to suggest possible industrial applications of this wine by-product to boost its use in a context of circular bioeconomy. 52 VOMs belonging to different chemical families were identified, namely alcohols (14), esters (14), carbonyl compounds (10), terpenoids (11), acids (2), and hydrocarbon (1), 18 of which were common to all GP analyzed. The contribution of esters and terpenoids to the total volatile profile is higher in GP from white grapes, whereas the remaining chemical families identified were higher in red grapes. In addition, the data obtained from antioxidant assays showed that GP could be considered a potential source of add-value compounds that might play a critical role in human health as free radical scavengers.

In addition, some esters (e.g., isoamyl acetate, hexyl acetate, ethyl hexanoate) and alcohols (e.g., benzyl alcohol, 2-phenethyl alcohol) identified in GP from the investigated grape varieties can be used as flavoring agents in various food products, as well as ingredients in perfumes, soaps, and other personal care products. Moreover, some of the terpenoids identified in GP have been reported as anti-inflammatory, antibacterial, antitumor, antiviral, and antioxidant agents, which gives the matrix potential use in the food, pharmaceutical and cosmetic industry, boosting the valorization of GP in the context of circular bioeconomy. Moreover, the data obtained will facilitate the projection and planning of the extraction in great quantity and industrial application of VOMs as flavoring and natural colorings in beverages productions (e.g. isoamyl acetate used as a flavoring agent in soft drinks); and pharmaceutical fields (e.g. ylangene is related to cytotoxic and antioxidant activities).

## CRediT authorship contribution statement

**Teresa Abreu:** Formal analysis, Investigation, Writing – original draft, preparation. **Gonçalo Jasmins:** Formal analysis, Investigation, Writing – original draft, preparation. **Catarina Bettencourt:** Formal analysis, Writing – original draft, preparation. **Juan Teixeira:** Conceptualization, Writing – review & editing. **José S. Câmara:** Conceptualization, Formal analysis, Writing – original draft, preparation, Writing – review & editing. **Rosa Perestrelo:** Conceptualization, Formal analysis, Supervision, Writing – original draft, preparation, Writing – review & editing, All authors have read and agreed to the published version of the manuscript.

## Declaration of competing interest

The authors declare that they have no known competing financial interests or personal relationships that could have appeared to influence the work reported in this paper.

## Data Availability

Data will be made available on request.
