# Supplementary material for: Tracing the volatilomic fingerprint of grape pomace as a powerful approach for its valorization
Source: Curr Res Food Sci. 2023 Sep 29;7:100608. doi: 10.1016/j.crfs.2023.100608 (PMC10570008; doi:10.1016/j.crfs.2023.100608)
Supplement: Multimedia component 1 [file mmc1.docx]

Figure S1 (Supplementary material) displays the typical chromatogram of the grape pomace obtained from a white (Boal) and red (Malvasia Roxa) *V. vinifera* L. grapes.

**Table S1:** Total phenolic content (TPC) and total flavonoid content (TFC) and antioxidant activities of GP from different *V. vinifera* L. grapes.

| **Grape pomace** | **TPC**  **(mg (GAE)/L)** | **TFC**  **(mg (GAE)/L)** | **ABTS**  **(mg (TRE)/L)** | **DPPH**  **(mg (TRE)/L)** |
| --- | --- | --- | --- | --- |
| Malvasia Roxa | 0.278 | 0.233 | 41.2 | 708.0 |
| Malvasia | 0.433 | 0.301 | 128.2 | 1112 |
| Boal | 0.460 | 0.347 | 244.6 | 1278 |
| Terrantez | 0.457 | 0.341 | 153.0 | 1163 |
| Verdelho | 0.745 | 0.558 | 257.4 | 3307 |
| Sercial | 0.716 | 0.693 | 256.3 | 3665 |
| Tinta Negra | 3.149 | 1.740 | 523.7 | 6446 |
| Complexa | 2.892 | 2.098 | 516.0 | 5695 |
